# Cryoablation of Early-Stage Primary Lung Cancer

**DOI:** 10.1155/2014/521691

**Published:** 2014-06-04

**Authors:** Masanori Inoue, Seishi Nakatsuka, Masahiro Jinzaki

**Affiliations:** ^1^Department of Radiology, Hiratsuka City Hospital, 1-19-1 Minamihara, Hiratsuka-shi, Kanagawa 254-0065, Japan; ^2^Department of Diagnostic Radiology, School of Medicine, Keio University, 35 Shinanomachi Shinjuku-ku, Tokyo 160-8582, Japan

## Abstract

Worldwide, lung cancer is the most commonly diagnosed cancer, and lobectomy is the gold-standard treatment for early-stage non-small cell lung cancer (NSCLC). However, many patients are poor surgical candidates for various reasons. Recently, image-guided ablation is being used for lung tumors. Cryoablation has been applied for the treatment of cancer in various nonaerated organs; recently it has been adapted to the treatment of lung tumors. Since an ice ball can be detected by computed tomography (CT), cryoablation of lung tumors is performed under CT guidance. Its first clinical application was reported in 2005, and it has been reported to be feasible in a few studies. Minor complications occurred at a high frequency (up to 70.5%), but major complications were rare (up to 1%). The most common complication is pneumothorax, and most cases need no further intervention. Local efficacy depends on tumor size and presence of a thick vessel close to the tumor. Midterm survival after cryoablation is 77%–88% at 3 years in patients with early-stage NSCLC. Although surgery is the gold-standard treatment for such patients, the initial results of cryoablation are promising. In this paper, the current status of cryoablation for primary lung tumors is reviewed.

## 1. Introduction

Worldwide, the leading causes of cancer-related deaths are primary and secondary lung tumors, with 1.59 million cases newly dying every year [1]. Surgical resection with lobectomy is the standard treatment for stage I non-small cell lung cancer (NSCLC), with proven long-term cure and survival [2–4]. However, over 20% of patients are not eligible for surgical intervention due to comorbidities or poor underlying lung reserve [5].

In patients who are unsuitable for surgery of both primary and metastatic lung tumors, image-guided thermal ablation is a rapidly advancing technique that has emerged as an alternative option [6, 7]. Accumulating evidence suggests that radiofrequency ablation (RFA) is a safe and feasible treatment option for the treatment of inoperable stage I NSCLC; however, there has been limited experience with cryoablation of NSCLC [8–14]. Since percutaneous cryoablation possesses several properties that make it an attractive ablation option, it has been applied for the treatment of cancer in various nonaerated organs [15, 16]. Such advantages include good visualization under computed tomography (CT) or magnetic resonance imaging guidance, preservation of collagenous architecture, and low intraprocedural pain [15]. One of the reasons why the use of cryoablation for lung tumors is limited is that the cryoprobes that were traditionally used were large (11 G; diameter = 3.0 mm) and had a blunt-tip. Therefore, percutaneous cryoablation for lung tumors has some difficulties, and adjunctive technique has been essential to penetrate small lung tumors [17, 18]. Currently, technological advances have allowed the development of cryoprobes of 17 G needles (diameter = 1.47 mm) and it has become easy to perform cryoablation of lung tumors [14, 19]. This paper will focus on the current status of cryoablation for lung tumors, especially for NSCLC, with regard to basic principles, feasibility, techniques, complications, and outcomes.

## 2. Basic Principles 

Liquid nitrogen-containing cryoprobes, which were developed by Cooper and Lee in the 1960s, have allowed deeper tissues to be treated with cryoablation [20, 21]. Due to the recent development of argon-based cryoablation systems, cryoablation probe diameters have decreased substantially, making percutaneous cryoablation more feasible [22]. Pressurized argon gas can cool to temperatures as low as −140°C, utilizing the Joule-Thomson effect. It has been commonly stated that −20°C is lethal for cells and that this temperature should be produced in the tissue to achieve a destructive effect; however, no in vivo experiments have been done to support the view that −20°C is an appropriate goal in cryoablation [23]. Thus, cryoablation uses lethal cold temperatures of −20°C to −40°C depending on tissue type [23, 24].

Cellular damage is the result of a complex combination of mechanisms in all sequences of the freeze-thaw cycle. Several mechanisms, including protein denaturation, cell destruction caused by osmotic shifts in intracellular and extracellular water, and tissue ischemia from microvascular thrombosis, are known to cause cellular injury in cryoablation [23]. Cellular injury mechanisms depend on four thermal parameters: cooling rate, end temperature, time held at the minimum temperature (or hold time), and thawing rate [24]. In general, rapid freezing, freezing to lower temperature, holding longer at the minimum temperature, and slow thawing, as well as repetition of the freeze-thaw cycle, increase cellular injury [24, 25]. As the temperature falls into the freezing range, ice crystal formation occurs first in the extracellular spaces, which causes dehydration of the cells. With further cooling, ice crystals may form within the cell. Intracellular ice formation is a serious threat to cell viability, disrupts organelles and cell membranes, and leads to cell death [24]. Furthermore, vascular stasis and thrombosis, following a cold injury, are also well known as another mechanism of cell injury in cryoablation. Vascular stasis may limit the thermal sink effect by occluding the pulmonary blood flow.

Currently, there are two commercially available percutaneous argon-based cryoablation devices: Cryohit (Galil Medical, Plymouth Meeting, PA) and Cryocare (Endocare, Irvine, CA). These systems can activate multiple thin-diameter probes simultaneously. Percutaneous cryoablation of lung tumors was performed under CT guidance because CT can detect lung tumors and parenchyma, allowing comparison of the rough ablative zone in relation to the tumor margins.

## 3. Technique

Percutaneous cryoablation of lung tumors is usually carried out with local anesthetics and conscious sedation under CT guidance. For curative cryoablation, the margins of the ice ball should extend 3 to 10 mm beyond the tumor margins [14, 19, 26]. In solid organ, ice ball can be clearly detected under CT guidance. However, the lung has a low water content, and the ablated area of lung parenchyma around the tumor, equal to margin of the ice ball, cannot be clearly visualized. Estimating whether the tumor has been ablated with sufficient treatment margins is therefore sometimes difficult on CT [27].

Wang et al. [17] first reported the clinical application of cryoablation for lung tumors in 2005. At that time, only 2.4 and 3 mm diameter probes were available, and it was difficult to penetrate the target tumor with the probes. Therefore, they inserted a 19 G needle through the tumor to the far margin at first and then advanced a dilator and 11 F sheath over the needle. Finally, after the needle and dilator were removed, a cryoprobe was introduced into the sheath. A treatment cycle consisted of 20-minute freezing followed by 10-minute thawing and then 20-minute refreezing.

In studies from the University of Keio [8, 18, 27–31], a 21 G guiding needle was inserted into the targeted tumor under intermittent 3-slice CT fluoroscopic guidance. Then, a modified coaxial system that consisted of an 8 or 11 G stainless-steel coaxial system consisting of an inner guiding sheath and an outer sheath was advanced over the guiding needle. After the inner sheath was removed, a 2.4 or 3.0 mm diameter cryoprobe was introduced into the outer sheath. Every procedure was performed using the triple freeze/thaw protocol. Freezing took 5 minutes for the first freeze and 10 minutes for the second and third freezes. Thawing with high-pressure helium gas was then performed until the temperature of the thermocouple in the cryoprobe reached 20°C.

Since Wang et al. [17] and the Keio university group [8, 18, 27–31] used the blunt-tip, 2.4 or 3.0 mm Endocare cryoprobes, placement of these probes required large introducer sheaths that may have also increased the risk of pneumothorax, as mentioned in the Complications section. More recently, 17 G thin cryotherapy needles became available, and Pusceddu et al. [19] reported cryoablation using thin needles. Cryoablation consisted of 2 cycles each of 12 min of freezing followed by a 4-min active thawing phase and a 4-min passive thawing phase for each one. A third freeze-thaw cycle was performed for the treatment of tumors located within 10 mm or in contact with the heart and major vascular structures.

Zhang et al. [14] also used 17 G thin cryotherapy needles. When there was the potential risk of position shift during puncture, a 21 G anchor needle was first used to fix the focus, and then two or three needles were inserted symmetrically into the edges of a tumor. For 17 G cryoablation needles, a tip-to-tip distance of 2 cm was normally applied so that a slight overlap of the ice balls created seamless cryoablation coverage. Two freeze-thaw cycles (15-min freeze, 3-min passive thaw) were used in all procedures.

## 4. Cryoablation Protocol

The number of freeze/thaw cycles differed among studies, and double or triple cycles were selected. In solid organs, double freeze/thaw cycles were usually adopted; however, it is reasonable to assume that the optimal protocol in an aerated tissue might differ from that in a solid tissue. According to a paper on the pathological changes after lung cryoablation reported by Izumi et al. [32], it appeared that infiltration of the blood from the frozen region into the aerated lung parenchyma during the first freezing had a profound effect on increasing thermal conductivity by pushing out the air. Nakatsuka et al. [33] showed in their animal experiment that the frozen area in the second cycle was dramatically enlarged as compared with the first cycle. They suggested that the first freezing cycle is just to create the optimal environment for heat conduction, the second is to produce a larger ice ball, and the third may be necessary for more effective cytotoxicity.

Hinshaw et al. [34] showed in an animal experiment that the triple-freeze protocol produces a zone of necrosis that is essentially identical to the double-freeze protocol despite a shorter overall freeze time (15 versus 20 min). They also noted that this property of the triple-freeze protocol could be exploited to create even larger zones of ablation or to shorten the overall procedure time.

## 5. Review of Studies on Cryoablation of Lung Tumors

A review of the literature written in English was conducted by searching the PubMed database using the keywords “cryoablation,” “lung,” and “cancer.” The publications cited by all electronically identified articles were further manually examined for potentially relevant studies. Clinical studies on cryoablation of lung cancer were selected. Case reports and reviews were excluded.

## 6. Outcomes

All data available on cryoablation of lung tumors come from observational studies, and the majority of these studies had a small sample size. Furthermore, there are few reports in the literature with an emphasis on stage I NSCLC. Thus, the literature has been summarized by tumor type in Table 1.

Wang et al. [17] reported their initial experience with percutaneous cryoablation of 234 tumors in 187 patients. They treated a mixed cohort of 196 primary and 38 metastatic lung cancers, achieving complete ice ball coverage of tumor in 98.7% and 87.2% for peripheral tumors smaller than 4 cm and larger than 4 cm, respectively. Tumor size and location were significant independent variables for tumor ice coverage. At 6 months, 86% of the treated tumors were stable or smaller than the original tumors on CT scans. The follow-up periods were too short to determine any survival benefit; however, palliative benefits of cryoablation were noted in terms of the Karnofsky Performance Status Scale and general health status.

In a lung cryoablation series involving 20 patients with 35 metastatic tumors (mean tumor size, 13.3 mm), Kawamura et al. [18] achieved an overall control rate of 80% and a 1-year survival rate of 89.4%, as determined by the Kaplan-Meier method. Zemlyak et al. [35] compared survival rates of patients undergoing sublobar resections (SLRs), RFA, and cryoablation for stage I NSCLC. There were 25 patients in the SLR group, 12 patients in the RFA group, and 27 patients in the PCT group. The overall 3-year survival rates for the SLR, RFA, and cryoablation groups were 87.1%, 87.5%, and 77%, respectively.

The 3-year cancer-specific and cancer-free survival rates for the SLR, RFA, and PCT groups were 90.6% and 60.8%, 87.5% and 50%, and 90.2% and 45.6%, respectively. Although there was a tendency toward higher cancer-free survival at 3 years for the SLR group (*P* > 0.05), they concluded that ablative therapies appear to be a reasonable alternative in high-risk patients not fit for surgery.

Yamauchi et al. [8] analyzed a sample of 22 patients with stage I NSCLC who were deemed medically inoperable. To the best of our knowledge, this is the first report that specifically focused on cryoablation in patients with medically inoperable stage I NSCLC. A total of 25 sessions for 34 tumors were performed. The size of tumors was 3 cm or less, with most 2 cm or less. The observation period ranged from 12 to 68 months (median 23 months). Local tumor progression after cryoablation was observed in one tumor (3%). The 2- and 3-year disease-free survival rates were 78% and 67%, respectively. Excellent overall survival rates of 88% at 2 years and 88% at 3 years were reported. These results are better than those previously reported for RFA. This was also presumably because, in their study, the tumors were 3 cm or less, whereas the previous RFA study included tumors that were 3-4 cm [9].

Zhang et al. [14] reported the results of 46 patients with NSCLC who were treated with cryoablation. Of the 46 patients with NSCLC, 12 had stage I NSCLC. The 2-year follow-up confirmed the survival of 43 patients. The 2-year overall survival in patients with stage I NSCLC was 100%. Based on the response evaluation criteria in solid tumors protocol (RECIST) criteria [36], complete response (CR) was achieved in 83.7% and partial response (PR) was achieved in 16.3%, with no cases of stable disease (SD) or progressive disease (PD).

Pusceddu et al. [19] reported the results of cryoablation using thin needles in 32 patients with 34 lung lesions (11 NSCLC, 23 metastases). Technical success (complete lack of enhancement) was achieved in 91% of treated lesions at 6-month CT follow-up.

Yamauchi et al. [28] treated 24 patients with 55 pulmonary metastases from colorectal cancer with cryoablation. The mean tumor diameter was 13 ± 7 mm (range, 3–31 mm). The median follow-up period was 40 months. The 1- and 3-year local progression-free rates were 90.8% and 59%, respectively. The 3-year local progression-free rates were 79.8% for tumors <15 mm in diameter and 28.6% (*P* = 0.001; log-rank test) for tumors >15 mm. The 1- and 3-year overall survival rates were 91% and 59.6%, respectively. They concluded that percutaneous cryoablation may have a useful role in the management of colorectal pulmonary metastases less than 15 mm in diameter when surgical resection is not an option.

Hashimoto et al. [29] compared the histologic findings in an animal experiment with CT findings immediately after cryoablation in clinical cases. They showed that a central solid zone and a surrounding air-containing zone on CT indicate complete tissue destruction and hemorrhage with air trapping, respectively. They also demonstrated that less than −20°C zone corresponds to the central solid zone on CT. They then extrapolated these results to local cancer control outcomes and showed that local cancer control was better in nodules contained within a central solid zone.

Yashiro et al. [27] reported their experience with cryoablation of 210 tumors (11 NSCLC, 199 metastases) in 71 patients in 102 sessions. This paper [27] provided important information about the rate of tumor progression and the risk factors for local progression after cryoablation of lung tumors. The median follow-up period was 454 (range, 79–2467) days. One-, two-, and three-year local progression-free rates were 80.4%, 69.0%, and 67.7%, respectively. On multivariate analysis, among the total of 210 tumors, larger tumor size (>20 mm) and presence of a thick vessel (diameter > 3 mm) close to the tumor were independent risk factors for local progression. Among the 167 tumors in which technical success, according to Hashimoto et al. [29], was achieved, existence of a thick vessel close to the tumor was found to be an independent factor for local progression on multivariate analysis.

There is accumulating evidence that RFA is a safe and feasible treatment option for the treatment of inoperable stage I NSCLC. The reported local control rates for RFA treatment of inoperable stage I NSCLC ranged from 58% to 69% [9–13]. Since tumor characteristics and follow-up periods were different, it is difficult to compare these results to those of cryoablation. However, initial experiences with cryoablation for early-stage NSCLC are promising [8, 14]. Most papers that focused on cryoablation in patients with metastatic lung tumors reported feasible results for local tumor control [18, 19, 27–29]. Although further accumulation of data regarding efficacy is necessary, cryoablation may be a feasible option in medically inoperable stage I NSCLC patients.

## 7. Imaging Evaluation after Cryoablation

Accurate imaging evaluation after cryoablation is challenging, because both a residual mass and an ablation zone are present, as compared to postsurgical follow-up. Clinically, local progression of tumors after cryoablation, which are supposed to be completely ablated based on images, is sometimes experienced. Therefore, imaging follow-up is indispensable to evaluate local recurrence after cryoablation. The assessment of response after thermal ablation is difficult because acute-phase reactions, such as alveolar hemorrhage, necrotic debris, inflammation, and edema around the target tumor, occur, and a scar persists after therapy [31].

There is considerable variation in how an ablated zone around the target tumor responds after treatment and progression occurs during follow-up. Ito et al. [31] examined the sequential change of ablation zone appearance after cryoablation and evaluated the size transition, shape transformation, enhancement, and other CT features. Compared with the previous image, all ablation zones showed significant enlargement on day 0 and size reduction at 1 month. At 1 and 3 months, all ablation zones showed rapid size reduction; then, at 6 months or later, the rate of size reduction decreased remarkably, which correlates well with previous reports [14, 17, 19]. They also classified the shape of the ablation zones after cryoablation into five patterns: a consolidation/atelectasis pattern, a nodular pattern, a stripe pattern, a pleural thickening pattern, and a disappearance pattern. The shape of the ablation zones tended to show the consolidation or nodular pattern within 1-week follow-up and size reduction and transformation into the stripe pattern at 1 month or later, and the ablation zones became indistinct later on. Atypical shape transformation indicated local progression. Those that reverse from a stripe pattern to a nodular pattern especially should be strictly followed up, because the majority of cases of local progression arose from the stripe pattern later than the 6-month follow-up after showing a shape transformation that did not conform to the typical tendency.

On follow-up contrast enhanced CT, both internal enhancement and marginal enhancement within the 3-month follow-up did not show a direct relationship with local progression. On the other hand, all internal enhancements after 6 months corresponded with local tumor progression.

The most common additional finding was peritumoral ground glass opacity, which was seen in 85% of the ablation zones. Cavitation was seen in 35% of ablation zones, and 96% of them disappeared within 6 months. A rim-like structure, which had a homogeneous wall with a thickness ranging from 1 mm to 5 mm, was often noted, especially on early follow-up images. Hashimoto et al. [29] suggested that ground glass opacity and rim-like structure correspond to severe pulmonary hemorrhage and extensive pulmonary edema, respectively.

Positron emission tomography (PET) might be more useful for evaluation, but its role has not yet been determined. As well as potential efficacy, PET might have a limitation because inflammation induced by cryoablation may result in false-positive results, especially in the early period after cryoablation [14].

## 8. Complications

In general, cryoablation appears to be a safe procedure with minimal morbidity and mortality [8, 14, 17–19, 27–30, 35]. Wang et al. [17], however, reported procedure-related mortality of 1.0% (2 of 187 patients). Causes of death were pulmonary embolus one day after cryoablation and acute respiratory distress syndrome one week later.

The most common complication encountered with cryoablation is pneumothorax. Pneumothorax occurs in approximately 12%–62% of patients after cryoablation, with approximately 0%–12% of pneumothoraces requiring chest tube insertion [14, 17, 19, 30, 35]. In a recent article, Inoue et al. [30] reported that a greater number of cryoprobes were associated with an increased risk of pneumothorax. In their study, the rate of pneumothorax was 62%, which is higher than in other papers [14, 17, 19, 30, 35]. They speculated that there are three possible reasons for the higher rate of pneumothorax in their study versus other studies: the number of cryoprobes, the thick modified coaxial system, and the modality used to detect pneumothorax [30]. The mean number of cryoprobes used in the present study was 2.4 ± 1.1, which is higher than in other studies. The ablation system they used was 8 or 11 G, which therefore may create a larger pleural hole. Although cryoprobes that were traditionally used were large (11 G; diameter = 3.0 mm), recent technological advances have allowed the development of thin needle probes (17 G; diameter = 1.47 mm) [19]. Therefore, this seems to be one of the reasons that the rate of pneumothorax after cryoablation has been decreasing in recent papers. Finally, Inoue et al. [30] detected minimal pneumothorax by CT scan. Minimal pneumothorax could not be detected by chest radiography.

Inoue et al. [30] also identified that male sex and no history of ipsilateral surgery were predictors for the need for chest tube insertion. Another common complication is pleural effusion, most cases of which are self-limiting, resolving with conservative management [30].

Reported rates of hemoptysis after cryoablation range from 0% to 62% [14, 17, 19, 30, 35]. All cases of hemoptysis in the previous study were self-limited. Herrera et al. [37] reported one case of death from massive hemoptysis after RF ablation of a centrally located lung tumor. A few studies have reported massive hemoptysis from a pulmonary pseudoaneurysm after lung RF ablation [38, 39]. The cause of the pseudoaneurysm may be thermal injury or direct puncture of the pulmonary artery. Thus, cryoablation is potentially safer than RF ablation in terms of thermal injury, because cryoablation preserves collagenous architecture.

A summary of reported complications following cryoablation is shown in Table 2. Other reported complications are cough, skin injury, arm paresis, temporary aphasia, phrenic nerve palsy, frostbite, empyema, tumor implantation, and subcutaneous emphysema.

## 9. Advantages and Disadvantages of Cryoablation

Percutaneous cryoablation is a minimally invasive alternative treatment. Cryoablation possesses several properties that make it an attractive ablation option. Such advantages include good visualization under CT guidance, preservation of collagenous architecture, and the capability to be performed under local anesthesia [30]. On the other hand, the major limiting factor of cryoablation is the size of the cryoablation zone and the thermal sink effect, which results in a higher local progression rate compared to surgical resection. As in any forms of thermal ablation, the size of tumors is one of the risk factors for local progression. Although the ablation zone of one cryoprobe is limited, multiple cryoprobes can be activated simultaneously, which enables creation of a bigger ice ball, and it may treat larger tumors. However, even in technically successful cases, in which the target tumor was covered by an ice ball with sufficient ablative margins, vessel proximity was a significant factor as a result of the thermal sink effect [27]. The flowing blood of the adjacent vessel prevents the temperature from decreasing to lethal levels. The lung receives all of the blood from the right side of the heart; therefore, this considerable blood flow causes a thermal sink effect. However, given that cryoablation is less invasive and can be performed repeatedly, repeat procedures can improve local control and overcome this drawback [40].

## 10. Conclusions

There is limited evidence for the possible use of cryoablation for early-stage NSCLC [8, 14, 19, 27, 35]. The 5 case series, including patients with NSCLC, in this present review were observational studies. These studies were very heterogeneous in terms of patient selection, cryoablation procedure, and tumor characteristics. Since only two studies reported estimated 3-year survivals, the therapeutic value of cryoablation has not yet been established. However, percutaneous cryoablation for lung tumor could be performed minimally invasively with acceptable complication rates. The early results of cryoablation for the treatment of patients with NSCLC appear feasible and encouraging, suggesting its potential to be one of the treatment options for patients who are unfit for surgery. Randomized trials comparing cryoablation with surgery are required.

## Figures and Tables

**Table 1:** The literature on cryoablation of lung tumors.

Study group and year	Number of patients	Tumor data	Tumor size* (mm)	Indications	Freeze/thaw cycle	Follow-up	LCR	Survival
Wang et al. [17], 2005	187: 165 NSCLC (5 stage I, 17 stage II, 80 stage III, and 63 stage IV), 22 metastasis	234 tumors: 196 primary cancer and 38 metastasis	43 ± 2 in peripheral locations and 64 ± 3 in central locations	Local control and palliation	Double	NA	NA	NA

Kawamura et al. [18], 2006	20: all metastasis	35	Mean tumor size, 13.3	Local control	Triple	9 to 28 months (median, 21 months)	LCR: 80%	1 y OS: 89.4%

Zemlyak et al. [35], 2010	27: all NSCLC (27 stage I)	27	NA	Local control	NA	Mean, 33 months	LCR: 89%	3 y OS/cancer-specific survival/cancer-free survival rate: 77.0%/90.2%/45.6%, respectively

Yamauchi et al. [28], 2011	24: all metastasis	55	13 ± 7	Local control	Triple	Median, 40 months	1 /3 y LCR: 90.8% and 59%, respectively, 3 y LCR of tumors <15 mm and >15 mm: 79.8% and 28.6%, respectively (*P* = 0.001)	1 /3 y OS: 91%/59.6%, respectively

Zhang et al. [14], 2012	46: all NSCLC (12 stage I, 19 stage II, and 15 stage III)	46	32 ± 11	Local control	Double	24 months	2 y LCR: 83.7%.	2 y OS: 93.5%.

Pusceddu et al. [19], 2013	32: 11 NSCLC (4 stage I, 3 stage II, 3 stage III, and 1 stage IV), 21 metastasis	34 tumors: 11 primary cancer and 23 metastasis	26 ± 12	Local control	Double or triple	6 months	1 /3 /6 mo technical success: 82%/97%/91%, respectively	NA

Yamauchi et al. [8], 2012	22: all NSCLC (22 stage I)	34	14 ± 6	Local control	Triple	12 to 68 months (median, 23 months)	LCR of 97%	2 /3 y OS: 88%/88%, respectively

Yashiro et al. [27], 2013	71 (patients characteristics were not reported)	210 tumors: 11 primary cancer and 199 metastasis	Mean tumor size, 12.8	Local control	Triple	79 to 2467 days (median, 454 days)	1 /2 /3 y LCR: 80.4%/69.0%/67.7%, respectively	NA

*Plus-minus values are means ± standard deviation, NA = not available, OS = overall survival, LCR = local control rate.

**Table 2:** Complications after cryoablation of lung tumors.

Study group and year	The diameter of probe or sheath	Pneumothorax (%)	Pneumothorax requiring chest tube insertion (%)	Hemoptysis (%)	Pleural effusion (%)	Fever (%)	Death (%)	Other complications
Wang et al. [17], 2005	A 11-F sheath was used	12.0	1.4	62.0	14.0	42 (<38.5°C)	1.0	Cough, skin injury, arm paresis, temporary aphasia, death, and subcutaneous emphysema

Zemlyak et al. [35], 2010	NA	37.0	NA	22.0	NA	NA	0	NA

Inoue et al. [30], 2012	An 8- or 11-G stainless-steel coaxial system was used	61.7	11.9	36.8	70.5	3.1 (<39.0°C)	0	Phrenic nerve palsy, frostbite, empyema, and tumor implantation

Zhang et al. [14], 2012	17 G cryotherapy needles (diameter = 1.47 mm)	19.6	4.4	39.1	NA	NA	0	NA

Pusceddu et al. [19], 2013	17 G cryotherapy needles (diameter = 1.47 mm)	21.0	0	0	NA	0	0	NA

NA = not available.
